# The extent of *androgen receptor* and *HER2* expression allows for targeted therapy in most cases of salivary duct carcinoma: analysis of clinical and histopathological data in a tertiary care center

**DOI:** 10.1007/s00405-024-08627-8

**Published:** 2024-04-08

**Authors:** Marcel Mayer, Philipp Wolber, Johanna Prinz, Louis Jansen, Julia Esser, Sami Shabli, Alexander Quaas, Jens Peter Klußmann, Shachi Jenny Sharma, Lisa Nachtsheim, Christoph Arolt

**Affiliations:** 1 Department of Otorhinolaryngology, Head and Neck Surgery, Medical Faculty, University of Cologne, Cologne, Germany; 2 Center for Molecular Medicine Cologne (CMMC), Medical Faculty, University of Cologne, Cologne, Germany; 3 Department I of Internal Medicine, Center for Integrated Oncology Aachen Bonn Cologne Duesseldorf, University of Cologne, Cologne, Germany; 4 Institute of Pathology, Medical Faculty, University of Cologne, Cologne, Germany

**Keywords:** Salivary gland carcinoma, Salivary duct carcinoma, Immunohistochemistry, Head and neck cancer, Targeted therapy, Survival, Androgen receptor, Human epidermal growth factor receptor 2

## Abstract

**Purpose:**

The incidence of salivary duct carcinoma (SDC) seems to be underestimated due to inaccurate classification. Further, the frequency of SDC patients with targeted therapy options according to current guidelines is unclear. Therefore, this study aimed at (a) describing the proportion of SDC among salivary gland carcinoma (SGC) before and after reclassification of cases initially classified as adenocarcinoma, not otherwise specified (ANOS); and (b) quantifying the frequency of SDC patients with targeted therapy options.

**Methods:**

All patients with SDC or ANOS treated in a tertiary care center between 1996 and 2023 were identified. Histopathological diagnosis was verified for patients primarily diagnosed with SDC and reviewed for patients initially diagnosed with ANOS. Clinical data for SDC patients were retrieved from clinical charts. Immunohistochemical (IHC) androgen receptor (AR) and HER2 staining was performed.

**Results:**

Among 46 SDC, 34 were primarily diagnosed as SDC and 12 had initially been classified as ANOS. The proportion of SDC among SGC was 12.1% and was rising when comparing the time periods 2000–2015 (7.1–11.5%) versus 2016–2023 (15.4–18.1%). Nuclear AR staining in > 70% of tumor cells was found in 56.8% and HER2 positivity (IHC 3 +) in 36.4% of cases. 70.5% of patients showed AR staining in > 70% of tumor cells and/or HER2 positivity and therefore at least one molecular target. 5-year overall and disease-free survival (DFS) were 62.8% and 41.0%. Multivariate Cox regression revealed positive resection margins (HR = 4.0, *p* = 0.03) as independent negative predictor for DFS.

**Conclusions:**

The results suggest a rising SDC incidence and show that the extent of the AR and HER2 expression allows for targeted therapy in most SDC cases.

## Introduction

Salivary gland carcinoma (SGC) is a rare malignant tumor of the head and neck representing a group of 21 entities with heterogenous clinical and biological characteristics [1]. Salivary duct carcinoma (SDC) is the entity with the worst prognosis, showing a 5-year overall survival (OS) of 40–65% [2–6] and a 4-/5-year disease-free survival (DFS) of 17–44% [2, 3, 5, 6]. Based on European cancer registry data, SDC only accounts for 4–5% of all SGC and 8–19% of all SGC are classified as adenocarcinoma, not otherwise specified (ANOS) (= salivary carcinoma, not otherwise specified (SCNOS) according to the 5th edition of the WHO classification) [1, 7, 8]. In a recent study 39%, of SGC that were initially diagnosed as ANOS were reclassified as SDC by pathologists with special expertise in SGC using contemporary immunohistochemical profiling and diagnostic criteria [9]. Therefore, it seems that the incidence of SDC may be largely underestimated in the cancer registry data, to date. As therapy strategies are depending on the SGC entity, the correct histopathological diagnosis is crucial for optimal therapeutic management. The ESMO guidelines on SGC recommend primary resection with ipsilateral neck dissection, followed by adjuvant radiation therapy for SDC [10]. In the recurrent/metastatic (R/M) situation, SDC patients should receive androgen deprivation therapy (ADT) in case of nuclear androgen receptor (AR) expression in > 70% of tumor cells, assessed by immunohistochemistry (IHC), or trastuzumab-based therapy in case of positivity of the human epidermal growth factor receptor 2 (HER2), defined by IHC score 3 + or fluorescence in situ hybridization (FISH) amplification [10]. Furthermore, retrospective data suggest a DFS benefit for SDC patients with AR expression in > 70% of tumor cells treated with adjuvant ADT together with radiation therapy compared to radiation therapy alone [11]. Although there is sufficient data showing that 69–100% of SDC patients are AR-positive and 13–71% are HER2-positive [12–16], literature is lacking an analysis of the frequency of cases with AR expression in > 70% of tumor cells. This study aimed at (a) describing the proportion of SDC among SGC before and after reclassification of cases initially classified as ANOS in the present collective and; (b) quantifying the frequency of SDC patients with targeted therapy options (AR expression in > 70% of tumor cells; HER2 positivity) by comprehensively analyzing clinical and histopathological data of SDC patients treated in a tertiary care center between 1996 and 2023.

## Methods

A retrospective clinical chart review was performed to identify all patients with the diagnosis of SDC and ANOS (= salivary carcinoma, not otherwise specified (SCNOS) according to the 5th edition of WHO classification for head and neck tumors [1]) treated at the Department of Otorhinolaryngology, Head and Neck Surgery of the University Hospital of Cologne, Germany, between January 1st, 1996 and December 31st, 2023. The histopathological diagnosis was verified for patients diagnosed with SDC and reviewed for patients initially diagnosed with ANOS with sufficient formalin-fixed paraffin-embedded (FFPE) material according to the 5th edition of WHO classification for head and neck tumors [1] defining SDC as mammary ductal carcinoma-like high grade carcinoma. Demographics, survival, and histopathological data for all patients with the diagnosis of SDC were extracted from the clinical and histopathological records. Regular post-treatment surveillance consisted of clinical examination including ultrasonography of the head and neck every 3 months for the first two years, every 6 months from the third to the fifth year, and every year after the fifth year. Additionally, head and neck MRI and CT scan of the thorax and upper abdomen were performed every 6 months for the first 2 years and every year from the third to the fifth year. In case of missing data on current tumor status within the clinical records, patients or their general practitioners were contacted to follow-up.

In case of missing AR or HER2 status, immunohistochemical staining was performed for all patients with sufficient FFPE material. Nuclear AR and membranous HER2 staining were assessed by two pathologists with special expertise in the field of SGC (CA, AQ). AR status was quantified by the percentage of tumor cells with nuclear AR expression. HER2 status was quantified using the breast scoring system endorsed by the College of American Pathologists, defining no staining or incomplete weak staining in ≤ 10% of tumor cells as 0, incomplete weak staining in > 10% of tumor cells as 1+, weak to moderate complete staining in > 10% of tumor cells as 2+, and complete strong staining in > 10% of tumor cells as 3 + [17].

The study was conducted in accordance with the Declaration of Helsinki and approved by the Ethics Committee of the University of Cologne (Approval code: 13-091).

Statistical analysis was performed using SPSS software version 28.0.0.0 (190) (IBM, Armonk, NY). Microsoft Excel spreadsheet was used for visualization of SDC proportions. The Kaplan–Meier method with 95% confidence intervals was used to test for OS, DFS, locoregional control (LRC), and distant control (DC) probability rates. OS was defined as the time interval between date of diagnosis and date of death. DFS was defined as the time interval between date of diagnosis and date of recurrence or death. LRC was defined as the time interval between date of diagnosis and date of locoregional recurrence. DC was defined as the time interval between date of diagnosis and date of distant recurrence. Cox proportional hazards survival regression was used to determine the influence of different variables on DFS and OS. Variables with significant association with survival in the univariate Cox regression analysis were tested in a multivariate Cox regression analysis for identification of independent prognostic factors. A *p*-value < 0.05 was considered statistically significant.

## Results

### Clinical data

Overall, 46 patients with SDC were included. Male gender was predominant (84.8%) and mean age at diagnosis was 66.7 (± 11.7) years. Most SDC were located in the parotid gland (93.5%). Primary therapy was surgery in 44 cases (95.7%) and radiotherapy with ADT in 2 cases (4.3%). Ipsilateral neck dissection was performed in most surgically treated patients (93.2%). 86.4% of surgically treated patients received adjuvant radiation therapy. Most surgically treated patients did not receive adjuvant systemic therapy (63.6%), while adjuvant chemotherapy, ADT, and trastuzumab were administered in 22.7%, 11.4%, and 2.7% of patients, respectively. In R/M SDC, chemotherapy, ADT, trastuzumab, and trastuzumab plus ADT showed clinical benefit rates (median duration of response) of 20.0% (21 months), 50.0% (22 months), 0.0%, and 100.0% (36 months) (Table 1).

**Table 1:** Clinical data

	All SDC n (%)
Demographics	
Female	7 (15.2)
Male	39 (84.8)
Age	66.7 ± 11.7
M-stage at diagnosis	
M0	44 (95.7)
M1	2 (4.3)
Localization	
Parotid gland	43 (93.5)
Submandibular gland	2 (4.3)
Minor salivary glands	1 (2.2)
Primary therapy	
Radiotherapy + ADT	2 (4.3)
Surgery	44 (95.7)
Submandibulectomy	2 (4.5)
Endoscopic endonasal	1 (2.3)
Parotidectomy	41 (93.2)
Subtotal	2 (4.9)
Total	15 (36.6)
Radical	24 (58.5)
Neck dissection	
Not performed	3 (6.8)
Selective/modified radical	33 (75.0)
Radical	8 (18.2)
N/A	2
Adjuvant radiotherapy	
No	6 (13.6)
Yes	38 (86.4)
N/A	2
Adjuvant systemic therapy	
Chemotherapy	10 (22.7)
ADT	5 (11.4)
Trastuzumab	1 (2.7)
None	28 (63.6)
N/A	2
Systemic therapy for R/M disease	
Chemotherapy	5
CBR	1 (20.0)
No response	4 (80.0)
Median duration of response	21 months
ADT	4
CBR	2 (50.0)
No response	2 (50.0)
Median duration of response	22 months
Trastuzumab	1
CBR	0
Trastuzumab + ADT	1
CBR	1 (100.0)
Median duration of response	36 months

ADT: androgen deprivation therapy; CBR: clinical benefit rate; R/M: recurrent/metastatic

### Histopathological data

The majority of patients (63.0%) showed advanced pathological T stage (T3/T4). Pathological N stage was positive in 79.5% of patients with a mean of 7.8 (± 11.5) lymph node metastases. 43.2% of patients had extracapsular spread. Perineural invasion was found in 78.0% and resection margins were free of tumor in 86.8% of cases. Nuclear AR staining in IHC was positive in 86.4% of cases with a mean AR expression in 63.1% of tumor cells. Nuclear AR staining in > 70% of tumor cells was found in 65.8% of AR-positive tumors and 56.8% of all SDC. HER2 status in IHC was 3 + in 36.4% of cases. Most HER2-positive cases (84.6%) showed homogenous strong membrane staining, while staining was heterogenous in the other cases. HER2 amplification status was available in 9 cases (2 IHC 0, 1 IHC 1+, 4 IHC 2+, 2 IHC 3+) and was positive in 1 IHC 3 + case. 70.5% of patients showed at least nuclear AR staining in > 70% of tumor cells or HER2 IHC 3 + and therefore at least one molecular target according to the ESMO guidelines [10]. 22.7% of patients showed two molecular targets with a nuclear AR staining in > 70% of tumor cells and HER2 IHC 3 + (Table 2).

**Table 2:** Histopathological data

	All SDC n (%)
Histopathological parameters T stage	
T1	5 (10.9)
T2	12 (26.1)
T3	14 (30.4)
T4	15 (32.6)
NA	2
N-stage	
N0	9 (20.5)
N +	35 (79.5)
N/A	2
Extracapsular extension	
ECE-	25 (56.8)
ECE +	19 (43.2)
N/A	2
Perineural invasion	
Pn0	9 (22.0)
Pn1	32 (78.0)
N/A	5
Resection status	
R0	33 (86.8)
R1	3 (7.9)
R2	2 (5.3)
N/A	8
AR/HER2 status	
AR status	
AR positivity	
No	6 (13.6)
Yes	38 (86.4)
N/A	2
Mean AR expression	(63.1)
AR expression > 70% (all SDC)	25 (56.8)
AR expression > 70% (AR + tumors)	25 (65.8)
HER2 status	
HER2 IHC	
0	11 (25.0)
1 +	7 (15.9)
2 +	9 (20.5)
3 +	16 (36.4)
N/A	2
HER2 amplification	
No	8 (88.9)
Yes	1 (11.1)
N/A	35
AR expression > 70% or/and HER2 3 +	31 (70.5%)
AR expression > 70% and HER2 3 +	10 (22.7%)

AR: androgen receptor; HER2: human epidermal growth factor receptor 2; IHC: immunohistochemistry

### Proportion of SDC

**Fig. 1:**
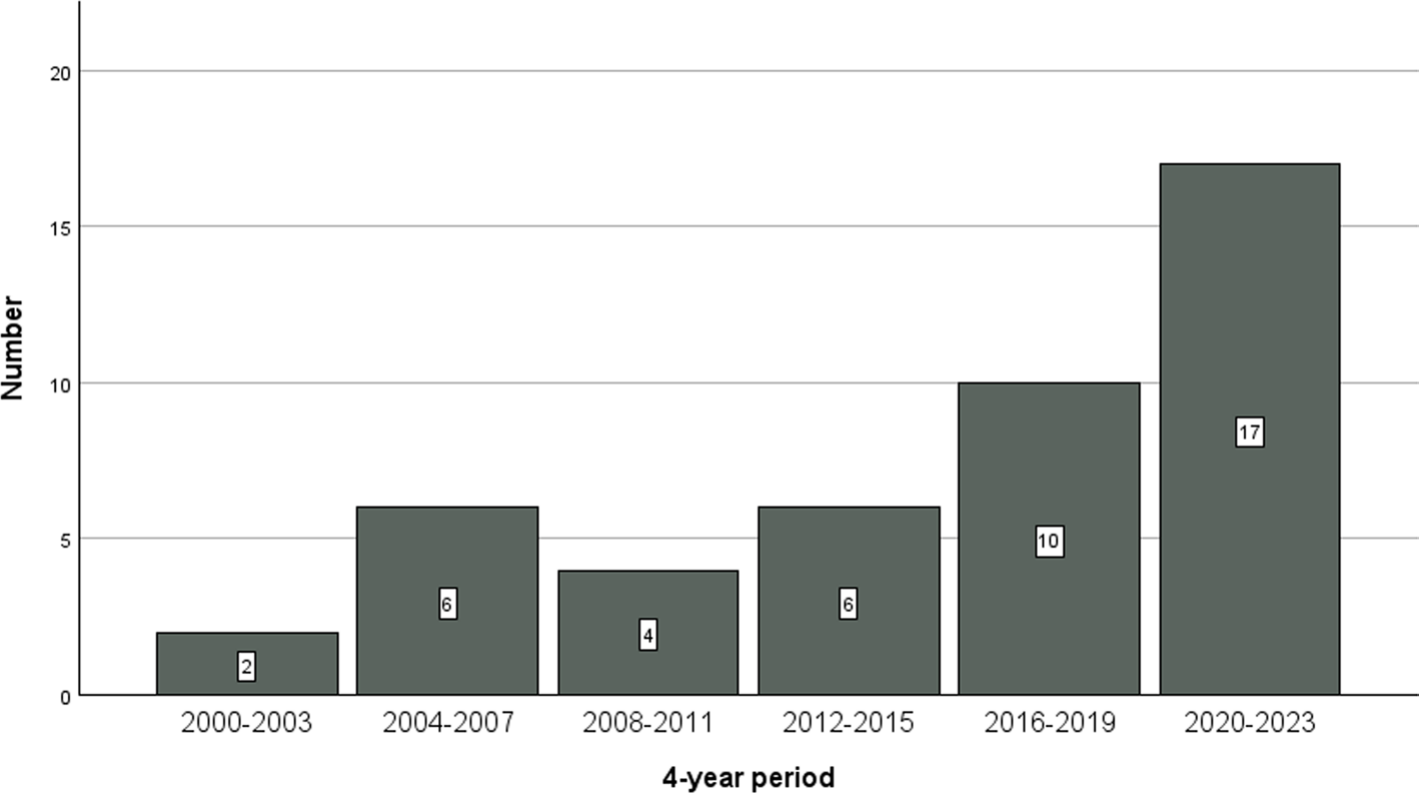
Absolute number of newly diagnosed salivary duct carcinomas per 4-year periods between 2000 and 2023

**Fig. 2:**
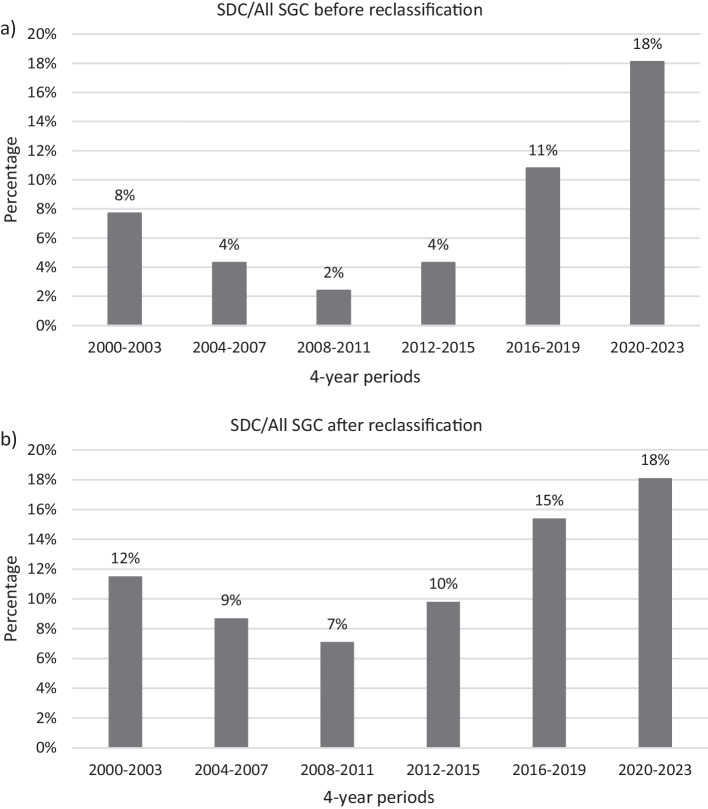
**a** Proportion of salivary duct carcinomas per all salivary gland carcinomas in 4-year periods between 2000 and 2023 before reclassification of adenocarcinomas, not otherwise specified, **b** after reclassification of adenocarcinomas, not otherwise specified. SDC: salivary duct carcinoma; SGC: salivary gland carcinoma

The single-center proportion of SDC among all SGC between 1996 and 2023 was 12.1% (46 out of 379 patients with primary SGC). Among patients with SDC, 34 were primarily diagnosed as SDC and 12 had initially been classified as ANOS. Figure 1 displays the rising absolute number of SDC between 2000 and 2023. Additionally, 12 SDC were diagnosed within the last two years. Figure 2 shows the proportion of SDC after and before reclassification of ANOS. For the time period from 2000 to 2019 (no cases reclassified after 2019) the proportion of SDC ranged between 2.4% and 10.8% before reclassification and between 7.1% and 15.4% after reclassification, respectively. After reclassification of ANOS there was still a rising SDC proportion when comparing the time periods 2000–2015 (7.1–11.5%) versus 2016–2023 (15.4–18.1%).

### Survival, locoregional/distant control and prognostic data

**Fig. 3:**
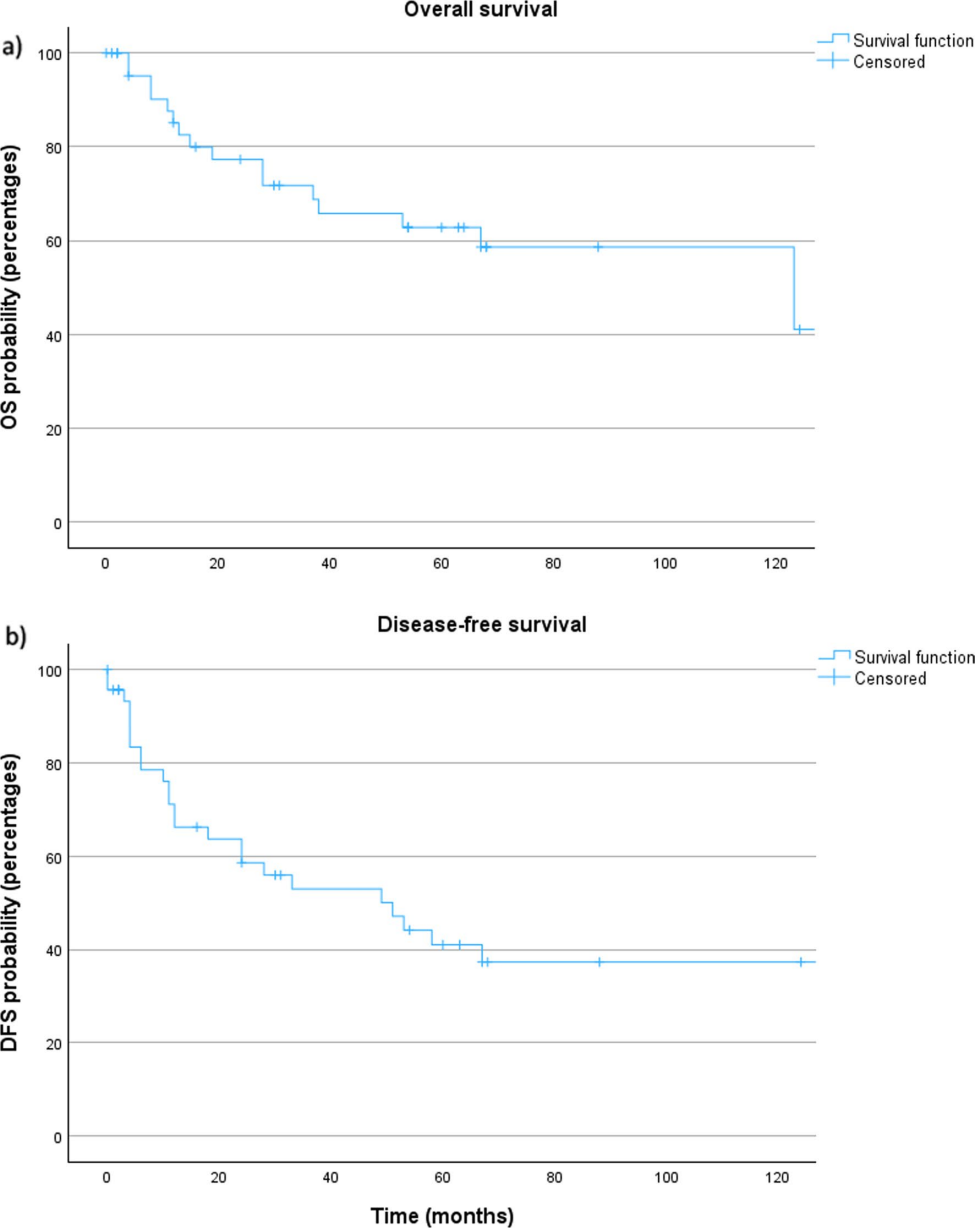
**a** Overall survival and **b** disease-free survival for all patients with salivary duct carcinoma. OS: overall survival; DFS: disease-free survival

**Fig. 4:**
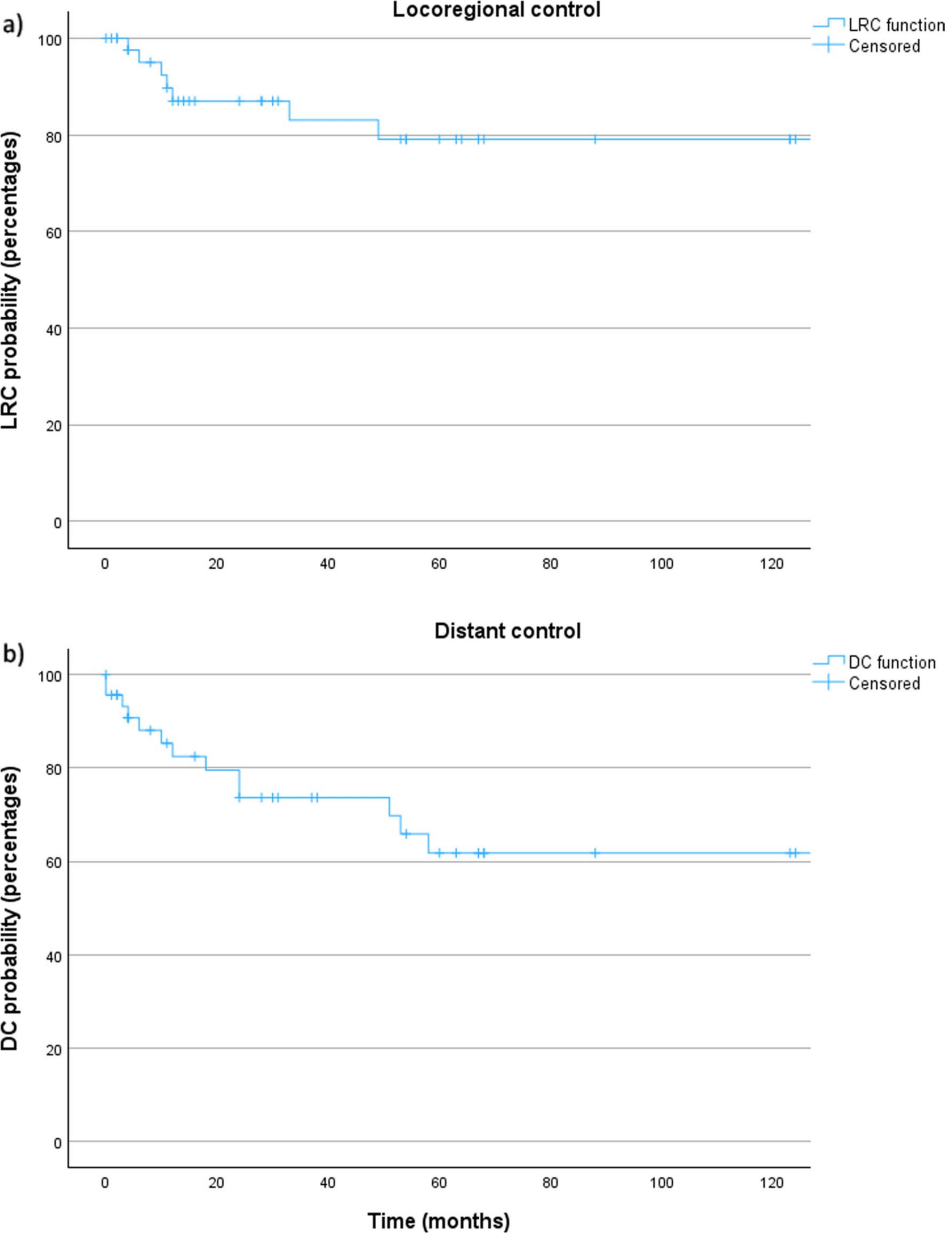
**a** Locoregional and **b** distant control for all patients with salivary duct carcinoma. *LRC: locoregional control; distant control*

The 5- and 10-year OS were 62.8% and 58.6% with a mean follow-up of 62.5 (± 70.9) months (Fig. 3a). The 5- and 10-year DFS were 41.0% and 37.3% (Fig. 3b). The 5-year locoregional control and distant control rates were 79.1% and 61.6%, respectively (Fig. 4). No locoregional or distant recurrence occurred after 5 years of follow-up.

Univariate Cox regression analysis revealed that pathological N + versus N − stage (HR = 10.4, *p* = 0.02) and positive versus negative resection margins (HR = 5.2, *p* < 0.01) were statistically significant negative predictors for DFS. Further, there was a statistical tendency for positive resection margins (HR = 2.9, *p* = 0.07) as a negative predictor for OS (Table 3). The multivariate model showed that positive versus negative resection margins (HR = 4.0, *p* = 0.03) was an independent negative predictor for DFS (Table 4).

**Table 3:** Univariate Cox regression analysis for prognostic value of clinicopathological variables on disease-free and overall survival

Variable	Disease-free survival			Overall survival		
	HR	CI	*p*-value	HR	CI	*p*-value
Age						
≤ 65	1.0			1.0		
> 65	0.75	0.3–1.7	0.48	0.76	0.3–1.9	0.56
Sex						
Male	1.0			1.0		
Female	1.0	0.3–2.9	0.99	1.3	0.4–4.1	0.63
pT-stage						
T1/2	1.0			1.0		
T3/4	1.6	0.7–3.7	0.28	1.9	0.7–5.4	0.22
N-stage						
N0	1.0			1.0		
N +	10.4	1.4–77.2	**0.02**	6.2	0.8–46.4	0.78
Perineural invasion						
Pn0	1.0			1.0		
Pn1	1.6	0.5–4.8	0.38	4.3	0.6–33.0	0.16
Extracapsular extension						
ECE-	1.0			1.0		
ECE +	1.7	0.7–4.0	0.21	1.4	0.5–3.7	0.54
Resection status						
R0	1.0			1.0		
R1/R2	5.2	1.5–17.4	** < 0.01**	2.9	0.9–9.4	0.07
AR positivity						
No	1.0			1.0		
Yes	0.9	0.4–2.8	0.91	0.8	0.2–2.7	0.68
HER2 positivity						
No	1.0			1.0		
Yes	0.9	0.4–2.4	0.84	1.0	0.3–2.8	0.97

AR: androgen receptor; HER2: human epidermal growth factor receptor 2; LN: lymph node(s). HR: hazard ratio; CI: 95% confidence interval; bold indicates statistically significant results

**Table 4:** Multivariate Cox regression analysis for prognostic value of clinicopathological variables on disease-free survival

Variable	Disease-free survival		
	HR	CI	*p*-value
N-stage (N0 vs. N +)	6.7	0.9–51.5	0.06
Resection status (R0 vs. R1/R2)	4.0	1.2–13.4	**0.03**

HR: hazard ratio; CI: 95% confidence interval

Bold indicates statistically significant results

## Discussion

A rising number of studies investigating SDC have been published within the last decade. Most of these either focused on clinical or immunohistochemical data. Recognizing different architectural patterns and rare subtypes [18], the diagnostic criteria of SDC have been formulated more widely in the more recent WHO classifications. Hence, from today’s perspective, the incidence of SDC was likely underestimated in the past [9]. Furthermore, improvement of immunohistochemical profiling and clinical studies evaluating targeted therapy options have led to a more precise indication for systemic therapy in the R/M situation [10]. Therefore, the present study is the first describing (a) the proportion of SDC among SGC before and after reclassification of cases initially classified as ANOS in a single-center series; and (b) the frequency of SDC patients with targeted therapy options according to current guidelines (AR expression > 70% in IHC for ADT; HER2 positivity for trastuzumab-based regimens).

A mean age at diagnosis of 66.7 (± 11.7) and a male predominance (84.8%) in the present series are consistent with previous data showing a mean/median age at diagnosis ranging between 60 and 68 years [2–4] and 72–83% of SDC patients being male [4, 5, 19]. While the literature shows that most SDC (72–83%) arise from the parotid gland [5, 16, 19, 20], predominance of parotid tumors was particularly high with 93.5% in this series, possibly due to this institution being a center for parotid gland surgery. As SDC is a biologically aggressive entity, advanced pathological T stage (T3/T4: 63.0%) and positive pathological regional lymph node status (79.5%) were to be expected and are consistent with previous data describing advanced T stage in 56–75% [3, 16] and pathological regional lymph node status in 68–82%% of cases [3, 16], respectively.

In accordance with the current ESMO guidelines for SGC [10], most tumor resections included ipsilateral neck dissection (92.9%) and most surgically treated patients received adjuvant radiation therapy (85.7%) in this series. Although cancer registry data, published in 2016, has shown that addition of adjuvant chemotherapy to radiation therapy does not improve OS of patients with high-risk SGC [21], 22.7% of patients in this series received adjuvant chemoradiation therapy. Most of these patients were treated before 2017 and the decision for addition of chemotherapy to adjuvant radiation therapy was made on case-by-case decisions after 2017. Nevertheless, the authors of this study strongly advocate for adhering to current guidelines and therefore avoiding adjuvant chemotherapy in SGC due to the side effects and missing evidence of survival benefits. Furthermore, recently published retrospective data suggest a significant survival benefit for SDC patients with AR expression in > 70% of tumor cells treated with adjuvant ADT (median duration of 12 months) together with radiation therapy compared to radiation therapy alone [11]. Based on this data, 5 recently diagnosed AR-positive patients with advanced tumor stages within the present series received adjuvant ADT together with radiation therapy on case-by-case decisions with the mean follow-up being too short to report sufficient results. Prospective clinical studies investigating the efficacy and safety of adjuvant ADT together with radiation therapy in SDC with a high extent of AR expression seem urgently needed to confirm an improvement of the outcome of these patients.

SDC is associated with an unfavorable survival due to a high rate of recurrence. 5-year OS and 4-/5-year DFS have been shown to range between 40–65% [2–6] and 17–44% [2, 3, 5, 6], respectively. A 5-year OS and a 5-year DFS of 62.8% and 41.0% in the present series are rather favorable compared to the existing literature. Previous studies have reported 5-year LRC and 5-year DC rates of 29–70% and 36–48% [2, 4, 5] being more unfavorable than the 5-year LRC and DC rates of 79.1% and 61.6% found in this study. A potential explanation is that more than one-fourth of the patients in this study were diagnosed between January 1st, 2022 and December 31st, 2023 and therefore have a follow-up of only up to 2 years. As shown in the existing literature, the results of the present study confirm a markedly higher LRC rate compared to the DC rate. This emphasizes the high relevance of systemic disease in SDC and therefore the necessity of effective systemic treatment options. Furthermore, neither locoregional, nor distant recurrence were found after a follow-up of 5 years in this study. Therefore, in contrast to adenoid cystic carcinoma showing a late recurrence after 5 years in 26% of cases [22], it seems that a follow-up of 5 years is sufficient for SDC.

Various independent negative prognostic factors on OS and/or DFS such as higher age, male gender, higher T/N stage, extracapsular spread, perineural invasion, facial nerve palsy, and postoperative radiation therapy have been identified in different studies [3, 16, 19, 23, 24]. This study is the first to reveal the independent negative prognostic influence of positive margins on DFS in SDC (HR = 4.0, *p* = 0.03), which seems plausible and has been proven for various other solid cancer entities [25–27]. Nevertheless, this result has to be emphasized as the surgical therapy of an SDC in the parotid gland poses a particular challenge due to the anatomical proximity to functionally relevant structures such as the facial nerve.

The overall proportion of SDC among primary SGC was 12.1% in the present series. This is markedly higher than the reported SDC proportion rates from European registries, being 5.4% in a Danish cohort of 1,601 patients treated between 1990 and 2015 and 3.5% in a German cohort of 1,680 patients treated between 2009 and 2018 [7, 8]. The main reason for this divergence may be underestimation of the incidence of SDC in cancer registry databases due to an inaccurate histopathological subclassification of SGC. More precisely, 39% of SGC, initially diagnosed as ANOS, were reclassified as SDC by pathologists with special expertise in SGC in a recent study using contemporary immunohistochemical profiling and diagnostic criteria [9]. Transferring this finding to the above-cited cancer registry databases (ANOS: 18.5% of all SGC in German registry and 8.4% in Danish registry) would result in an SDC proportion of around 10% of all SGC in these studies. These results underline the importance of accurately reviewing cases of ANOS by a pathologist specialized in head and neck pathology, especially in the context of established targeted therapies for SDC in the R/M setting and emerging evidence for targeted therapies in the adjuvant situation. Moreover, the present study also shows a rising proportion of SDC after reclassification of cases initially classified as ANOS. While SDC accounted for 7–12% of all primary SGC between 2000 and 2015, 15–18%% of all SGC diagnosed between 2016 and 2023 were SDC. If these data reflect a rise of the SDC incidence also outside of this single-center institution is unclear and needs further research. An accurate histopathological review of cases initially diagnosed as ANOS in large cohorts—ideally in cancer registry databases—of SGC is needed to answer this question. As the median age at diagnosis of SDC compared to other SDC entities is relatively high with up to 68 years [4], an increasing life expectancy in Europe during the last decades [28] may serve as an explanation for the observed rising SDC proportion.

Although various studies have shown that 69–100% and 13–71% of SDC are AR-positive and HER2-positive [12–16], respectively, the present study is the first to evaluate the frequency of SDC patients with established targeted therapy options according to current guidelines. The ESMO guidelines on SGC recommend ADT in case of AR expression in > 70% of tumor cells and trastuzumab-based therapy in case of HER2 positivity in R/M SDC [10]. 56.8% of all SDC in this series showed nuclear AR expression in > 70% of tumor cells. Moreover, 36.4% of all SDC were positive for HER2 (IHC 3 +). This resulted in 70.5% of patients being either eligible for targeted ADT or trastuzumab-based therapy.

Importantly, expression of AR and HER2 was not mutually exclusive as around one-fourth of cases showed AR expression in > 70% of tumor cells and positivity for HER2 (IHC 3 +). This is particularly important as a crosstalk between the AR and HER2 pathways has been shown for prostate carcinoma [29–32]. In detail, the activation of the HER2 pathway is inhibited by prostate-specific cPAcP in androgen-dependent prostate cancer. Androgen decreases cPAcP resulting in HER2-activated tyrosine phosphorylation of p38-MAPK and ERK1/2 which in turn lead to cell proliferation via the intranuclear androgen-response element. Loss of cPAcP leads to androgen-independent prostate cancer cell proliferation in androgen-independent prostate cancer cells [29, 30]. Crosstalk between the AR and HER2 pathways is also suspected in SDC as patients treated with enzalutamide with AR + /HER2 + tumors experienced a lower clinical benefit rate (22.2%) than patients with AR + /HER2 − tumors (45.8%; *p* = 0.013) in a phase II trial [33]. Therefore, combination therapies simultaneously targeting the AR and HER2 pathways in SDC may improve patient outcomes in the future.

This study has several limitations. First, clinical data were collected retrospectively. Further, no sufficient FFPE tissue for IHC was available in 2 cases of SDC. Lastly, as FISH results evaluating the HER2 amplification status were only available in 9 cases, HER2 positivity may be underestimated.

Overall, this study displays the proportion of SDC among SGC in a single-center before and after reclassification of cases initially classified as ANOS and suggests a rising absolute and relative incidence of SDC within the last years. Moreover, the results show that the extent of AR and HER2 expression allows for established targeted therapy according to the current guidelines in most cases of SDC and that AR and HER2 expression are not mutually exclusive.

## Data Availability

The datasets generated and analyzed during the current study are available from the corresponding author on reasonable request.
